# Exploration of prognostic factors for prediction of mortality in elderly CAP population using a nomogram model

**DOI:** 10.3389/fmed.2022.976148

**Published:** 2022-10-10

**Authors:** Chunxin Lv, Mengyuan Li, Wen Shi, Teng Pan, Abdul Muhith, Weixiong Peng, Jiayi Xu, Jinhai Deng

**Affiliations:** ^1^Department of Oncology, Punan Hospital of Pudong New District, Shanghai, China; ^2^Faculty of Life Sciences and Medicine, School of Cancer and Pharmaceutical Sciences, King’s College London, London, United Kingdom; ^3^Department of Dermatology, Punan Hospital of Pudong New District, Shanghai, China; ^4^Key Laboratory of Cancer Prevention and Therapy, The Third Department of Breast Cancer, Tianjin’s Clinical Research Center for Cancer, National Clinical Research Center for Cancer, Tianjin Medical University Cancer Institute and Hospital, Tianjin, China; ^5^Department of Oncology, Royal Marsden Hospital, London, United Kingdom; ^6^Hunan Zixing Artificial Intelligence Technology Group Co., Ltd., Changsha, China; ^7^Department of Geriatric, Minhang Hospital, Fudan University, Shanghai, China; ^8^Richard Dimbleby Department of Cancer Research, Comprehensive Cancer Centre, King’s College London, London, United Kingdom

**Keywords:** CAP in elderly patients, nomogram model, prognosis, CURB-65, qSOFA

## Abstract

**Background:**

The incidence and mortality rate of community-acquired pneumonia (CAP) in elderly patients were higher than the younger population. The assessment tools including CURB-65 and qSOFA have been applied in early detection of high-risk patients with CAP. However, several disadvantages exist to limit the efficiency of these tools for accurate assessment in elderly CAP. Therefore, we aimed to explore a more comprehensive tool to predict mortality in elderly CAP population by establishing a nomogram model.

**Methods:**

We retrospectively analyzed elderly patients with CAP in Minhang Hospital, Fudan University. The least absolute shrinkage and selection operator (LASSO) logistic regression combined with multivariate analyses were used to select independent predictive factors and established nomogram models *via* R software. Calibration plots, decision curve analysis (DCA) and receiver operating characteristic curve (ROC) were generated to assess predictive performance.

**Results:**

LASSO and multiple logistic regression analyses showed the age, pulse, NLR, albumin, BUN, and D-dimer were independent risk predictors. A nomogram model (NB-DAPA model) was established for predicting mortality of CAP in elderly patients. In both training and validation set, the area under the curve (AUC) of the NB-DAPA model showed superiority than CURB-65 and qSOFA. Meanwhile, DCA revealed that the predictive model had significant net benefits for most threshold probabilities.

**Conclusion:**

Our established NB-DAPA nomogram model is a simple and accurate tool for predicting in-hospital mortality of CAP, adapted for patients aged 65 years and above. The predictive performance of the NB-DAPA model was better than PSI, CURB-65 and qSOFA.

## Introduction

Community-acquired pneumonia (CAP) is the leading cause of mortality and morbidity with substantial clinical and economic impact, especially in the elderly population (1, 2). Older patients (aged ≥ 65) hospitalized with CAP are at high risk for incidence and mortality due to several intrinsic factors, including comorbidities, nutritional status, weakened immune system (3–6). Specifically, it has been reported that the risk of in-hospital mortality for elderly with CAP was 19.8% much higher than that of younger population with a percentage of 8.4% (7). Thus, for a better health service and to decrease the in-hospital mortality, more attention should be paid on elderly patients aged 65 years and above with CAP (8).

In clinic, several assessment tools for CAP patients have been developed and applied in early detection and assessment of high-risk patients, including quick Sequential Organ Function Assessment (qSOFA), Pneumonia Severity Index (PSI), and Combination of Confusion, Urea, Respiratory Rate, Blood Pressure, and Age ≥ 65 (CURB-65) (9–11). These score systems are widely used in predicting the prognosis of patients with CAP for different reasons. CURB-65 and qSOFA are applied due to advantages of being concise and easy-to-use (12–14), and PSI for its capacity with high sensitivity for mortality prediction (15). However, several disadvantages exist to limit the efficiency of these tools for accurate assessment, especially for elderly CAP. For example, some essential parameters, like a variety of comorbidities and weakened immune system, malnutrition, and swallowing dysfunction which elderly patients usually suffer from, are not included in available validated pneumonia severity scores (3, 16). Consequently, these score systems demonstrated poor performance in predicting pneumonia mortality of patients aged over 65, supported by previously reported study by Carmo et al. showing the AUC of CURB-65 and qSOFA were only 0.65 and 0.64, respectively, in predicting the mortality of elderly patients with a median age of 81 years (IQR,67–90) (17).

Therefore, we aimed to explore a comprehensive and easier-to-use tool to predict mortality in elderly CAP population by using a nomogram model. Further analyses showed our new nomogram model presented superiority to PSI, CURB-65, and qSOFA in predicting the prognosis of the elder patients with CAP in our study cohorts.

## Materials and methods

### Study population

This is a retrospective cohort study conducted in the Minhang Hospital, Fudan University in Shanghai from 1 January 2020 to 1 September 2022. It was approved by the Ethics Committee of the Minhang Hospital, Fudan University in Shanghai [Lot No: Medical Ethics Committee (2017) No. 42]. Regarding the patient informed consent statement, we notified patients and/or their legal guardians by telephone and asked for consent. Written informed consents were sent to patients and/or their legal guardians who agreed to participate in the study for signature. Signatures of study population were obtained and all procedures are in accordance with the Declaration of Helsinki. Inclusion criteria were as follows: (1) Age ≥ 65 years; and (2) Diagnosed with CAP community onset and respiratory symptoms compatible with pneumonia with new infiltrates on chest X-ray or CT scan. The exclusion criteria were as follows: (1) Immunosuppression, such as in the course of corticosteroids (> 14 days), HIV-positive, chemotherapy or radiotherapy within 90 days and transplant recipients; (2) Living in Nursing home.

### Data collection

Electronic medical records were retrospectively reviewed to obtain the data including age, gender, comorbidities, hospitalization days, consciousness state, vital signs, dysphagia and laboratory variables within 24 h of admission from the electronic medical records in Minhang Hospital, Fudan University. Aforementioned variables were used to determine PSI, qSOFA, CURB-65 scores were calculated according to the physiological and laboratory variables.

In details, variables used in CURB-65 score assessment: confusion, urea > 7 mmol/L, respiratory rate ≥ 30/min, blood pressure (systolic blood pressure < 90 mmHg or diastolic blood pressure ≤ 60 mmHg) and age ≥ 65 years; variables used in qSOFA score assessment: systolic blood pressure ≤ 100 mmHg, respiratory rate ≥ 22/min, and altered cognitive state; and variables used in PSI score assessment: demographics, comorbidities, a physical examination, and laboratory and radiological findings.

### Statistical analysis

Statistical analysis was executed with R (version4.1.1). All the hypothesis tests were two-sided with a significance level of 0.05. We used Student’s *t*-test to compare continuous variables, and the chi-square test to compare distributed variables. Numerical parametric data were described as mean (*SD*), other continuous non-parametric data were described as median (inter-quartile range) and classification variables were reported as percentages. We used the least absolute shrinkage and selection operator (LASSO) logistic regression combined with multivariate analyses to select independent predictive factors and established nomogram models *via* the rms package in R software. The c-index was calculated to quantify the predictive accuracy of the nomograms. Calibration plots were generated to check the consistency between the predicted and observed probabilities. Decision curve analysis (DCA) was employed to examine the clinical net benefit of a predictive model by the rmda package in R software. Finally, receiver operating characteristic curve (ROC) was used to compare the performance of the nomograms with CURB-65 and qSOFA in predicting the mortality of elderly patients with CAP.

## Results

### Clinical characteristics of patients

**FIGURE 1:**
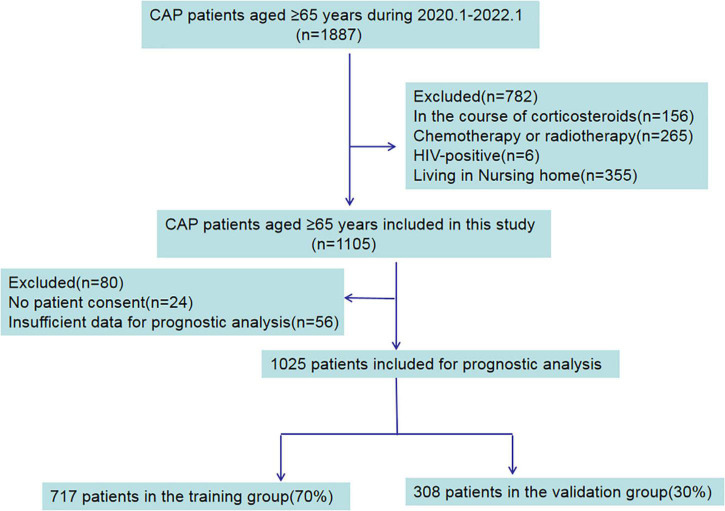
Study flowchart.

In total, 1887 elderly patients diagnosed with CAP were collected from the electronic medical records and recruited in this study. 782 patients were excluded for being on a course of corticosteroids, HIV-positive, chemotherapy or radiotherapy and living in nursing homes. In the remaining 1,105 patients, 24 patients with no patient consent, and 56 patients with insufficient data were excluded. The flow chart used for data selection is shown in Figure 1. Ultimately, 1,025 patients were selected for this study including 569 males and 456 females, aged 79.8 ± 8.8 years old. According to the available survival data, there were 895 survivor cases and 130 non-survivor cases included. The total hospital mortality rate was recorded as 12.7% (130/1,025). Through the basic characteristics analyses, we observed that there were significant differences between survivor groups and non-survivor groups regarding age, pulse, systolic pressure, diastolic pressure, leucocyte count, neutrophil count, lymphocyte count, NLR (neutrophil-lymphocyte ratio), platelet count, c-reactive protein (CRP), procalcitonin (pct), albumin, prealbumin, urea nitrogen (BUN), D-dimer, low-density lipoprotein (*p* < 0.05) (Table 1).

**TABLE 1:** Basic characteristics of survival and death patients.

Predictive factors	Survivor groups (*n* = 895)	Non-survivor groups (*n* = 130)	*P-value*
Gender (male/female)	493/402	76/54	0.468
Age (years)	79.2 ± 8.8	84.1 ± 8.1	<0.0001
Smoking	160/895	21/130	0.627
Pulse (/min)	86.2 ± 15.2	95.1 ± 20.6	<0.0001
Systolic pressure (mmHg)	128.6 ± 19.2	123.7 ± 34.8	0.03
Diastolic pressure (mmHg)	73.9 ± 11.1	68.4 ± 19.5	<0.0001
Respiratory rate (/min)	19.6 ± 2.9	19.4 ± 2.5	0.32
Dysphagia	135/895	19/130	0.89
Leucocyte count (×10^9^)	8.9 ± 4.3	12.2 ± 5.7	<0.0001
Neutrophils count (×10^9^)	7.0 ± 5.0	10.5 ± 5.6	<0.0001
Lymphocyte count (×10^9^)	1.3 ± 0.8	0.9 ± 0.8	<0.0001
NLR	7.6 ± 8.2	17.9 ± 14.6	<0.0001
Platelet count (×10^9^)	227.2 ± 95.7	201.4 ± 106.5	0.003
CRP (μg/ml)	69.1 ± 66.6	127.5 ± 89.2	<0.0001
pct (ng/ml)	1.7 ± 8	8.1 ± 19.8	<0.0001
Albumin (g/L)	33.6 ± 5.2	27.8 ± 4.9	<0.0001
Prealbumin (mg/L)	123.7 ± 57.0	106.5 ± 53.3	0.0008
Low-density lipoprotein(mmol/L)	2.3 ± 0.9	2.0 ± 0.9	0.0009
BUN (mmo/l)	6.0 ± 4.9	15.8 ± 11.1	<0.0001
D-dimer (mg/L)	2.6 ± 5.2	7.7 ± 11.5	<0.0001
Electrolyte disturbance	186/895	23/130	0.41
Cancer	50/895	11/130	0.22
Chronic kidney disease	78/895	12/130	0.85
Congestive heart failure	134/895	19/130	0.91
Cerebrovascular disease	219/895	35/130	0.55
Coronary heart disease	144/895	20/130	0.83
Hypertension	361/895	60/130	0.21
Diabetes	196/895	23/130	0.26

NLR, neutrophil-lymphocyte ratio; CRP, c-reactive protein; pct, procalcitonin; BUN, blood urea nitrogen.

### Development of nomogram for mortality prediction

Next, to better establish an assessment model, we developed a nomogram model for predicting in-hospital mortality of elderly patients with CAP. The patients were randomly divided into the training cohort (*n* = 717) and the validation cohort (*n* = 308). No statistically significant difference was observed in baseline characteristics between these two study groups (Table 2).

**TABLE 2:** Characteristics of the training and validation group.

Predictive factors	Training group (*n* = 717)	Validation group (*n* = 308)	*P-value*
Survivor/Death	624/93	271/37	0.67
Gender (male/female)	387/330	182/126	0.13
Age (years)	79.8 ± 8.8	79.8 ± 8.9	0.98
Smoking	123	60	0.46
Pulse (/min)	87.8 ± 16.5	86.1 ± 15.6	0.13
Systolic pressure (mmHg)	127.7 ± 21.9	128.5 ± 21.9	0.60
Diastolic pressure (mmHg)	73.1 ± 12.7	73.6 ± 12.5	0.53
Respiratory rate (/min)	19.6 ± 3.2	19.5 ± 1.6	0.52
Dysphagia	101	53	0.27
Leucocyte count (×10^9^)	9.2 ± 4.6	9.5 ± 4.7	0.41
Neutrophils count (×10^9^)	7.3 ± 4.5	7.7 ± 6.7	0.15
Lymphocyte count (×10^9^)	1.2 ± 0.8	1.3 ± 0.8	0.36
NLR	8.7 ± 9.1	9.3 ± 11.5	0.34
Platelet count (×10^9^)	220.6 ± 100.1	231.6 ± 90.6	0.10
CRP (μg/ml)	76.7 ± 73	76.2 ± 71.3	0.93
pct (ng/ml)	2.7 ± 11.3	2.2 ± 8.4	0.48
Albumin (g/L)	32.8 ± 5.5	32.9 ± 0.32	0.74
Prealbumin (mg/L)	121.7 ± 55.9	121 ± 58.6	0.86
Low-density lipoprotein (mmol/L)	2.3 ± 0.9	2.3 ± 0.9	0.15
BUN (mmo/l)	8.1 ± 6.8	7.8 ± 6.6	0.40
D-dimer (mg/L)	3.3 ± 6.8	3.2 ± 5.8	0.81
Electrolyte disturbance	141	68	0.47
Cancer	48	13	0.13
Chronic kidney disease	68	22	0.26
Congestive heart failure	103	50	0.51
Cerebrovascular disease	173	81	0.57
Coronary heart disease	113	51	0.79
Hypertension	296	125	0.89
Diabetes	158	61	0.52

NLR, neutrophil-lymphocyte ratio; CRP, c-reactive protein; pct, procalcitonin; BUN, blood urea nitrogen.

**FIGURE 2:**
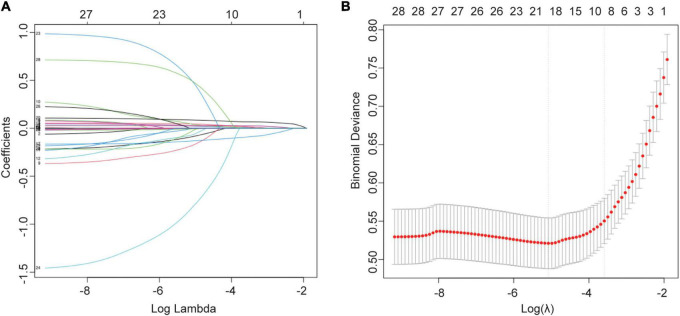
Selection of variables for mortality were performed using the LASSO analysis. **(A)** Model coefficient trendlines of the 28 variables for mortality. The profile graph was plotted by coefficients against the L1 norm. **(B)** Tuning parameter λ in the LASSO model. The parameter (λ = 0.068) was selected under the minimum criteria. The vertical line was drawn at the value selected by 10-fold cross-validation, including optimized 8 non-zero coefficients.

**FIGURE 3:**
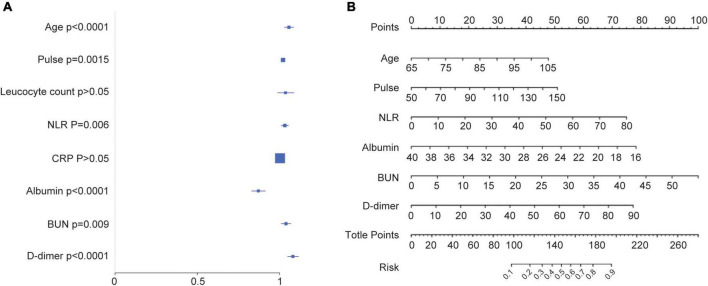
Establishment of a nomogram model for predicting the mortality of elderly patients with CAP. **(A)** Forest map showing multivariate analyses. **(B)** The nomogram model for predicting the mortality of elderly patients with CAP. The nomogram factors included age, pulse, NLR, albumin, BUN, and D-dimer. The nomogram summed the scores for each scale and variable. The total score on each scale indicated the risk of mortality. NLR (neutrophil-lymphocyte ratio), BUN (blood urea nitrogen).

In the training cohort, we applied all 28 potential predictive factors collected for the LASSO analysis, including age, gender, vital signs, routine blood analyses, comorbidities and other variables listed in Table 1. From the analysis, the most appropriate tuning parameter λ for LASSO regression was 0.010, where the partial likelihood binomial deviance reached its minimum value, and 8 variables with non-zero coefficients were retained in the LASSO analysis (Figures 2A,B). Then these 8 variables were used for multiple logistic regression analysis. Among them, 6 variables were demonstrated to be independently and statistically significant predictors, including age, pulse, NLR, albumin, BUN, and D-dimer. The results of the LASSO analysis and multiple logistic regression analysis are presented in Table 3. And the forest plot was showed in Figure 3A. Then these 6 variables were used for the establishment of a nomogram model, which facilitates the prediction of mortality of elderly patients with CAP, named as NB-DAPA nomogram (Figure 3B). Among these parameters, two factors, albumin and BUN, showed the best predictive capabilities (Figure 3B).

**TABLE 3:** LASSO analysis and multiple logistic regression analysis.

Predictive factors	Lasso_default	Lasso_lowestλ	Lasso_lowestSE	Multivariate analysis		
				OR	95% CI	*P-value*
**Gender**						
Age	0.044823979	0.044823979	0.014233861	1.057	1.029–1.086	<0.0001
**Smoking**						
Pulse	0.021976568	0.021976568	0.006401775	1.021	1.008–1.034	0.0015
Systolic pressure	.	.	.			
Diastolic pressure	–0.003356985	–0.003356985	.			
Respiratory rate	–0.086546902	–0.086546896	.			
Dysphagia	–0.177767796	–0.177767783	.			
Leucocyte count	0.040249303	0.0402493	0.002161209	1.036	0.987–1.088	0.153
Neutrophils count	–0.001116826	–0.001116825	.			
Lymphocyte count	–0.067477336	–0.067477324	.			
NLR	0.027343484	0.027343485	0.026505547	1.032	1.009–1.055	0.006
Platelet count	–0.000652715	–0.000652714	.			
CRP	0.002403924	0.002403924	0.000865519	1.003	0.999–1.006	0.09
pct	–0.006070107	–0.006070106	.			
Albumin	–0.131650928	–0.131650927	–0.091717953	0.871	0.830–0.914	<0.0001
Prealbumin	.	.	.			
Low-density lipoprotein	.	.	.			
BUN	0.086045012	0.086045011	0.067554087	1.039	1.009–1.069	0.009
D-dimer	0.027053928	0.027053928	0.016706245	1.081	1.049–1.115	<0.0001
Electrolyte disturbance	–0.026370334	–0.02637032	.			
Cancer	0.487694185	0.487694147	.			
Chronic kidney disease	–0.81974051	–0.81974048	.			
Congestive heart failure	.	.	.			
Cerebrovascular disease	.	.	.			
Coronary heart disease	.	.	.			
Hypertension	0.489709721	0.489709701	.			
Diabetes	.	.	.			

LASSO, least absolute shrinkage and selection operator; NLR, neutrophil-lymphocyte ratio; CRP, c-reactive protein; pct, procalcitonin; BUN, blood urea nitrogen.

### Performance and validation of nomogram for mortality prediction

**FIGURE 4:**
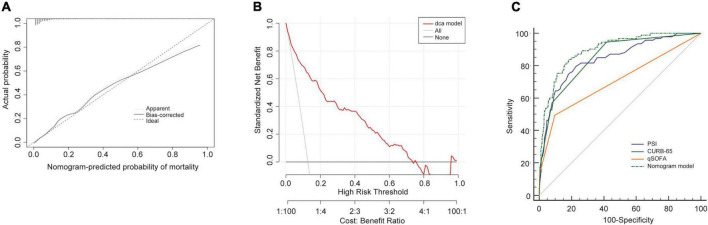
Performance of the nomogram model in training cohort. **(A)** Calibration curves for predicting the mortality in the training set. **(B)** Decision curve analysis for the predict-ed-nomogram model of mortality in the training set. **(C)** The receiver operator characteristic curves (ROC) of the nomogram model, PSI, CURB-65 and qSOFA in training cohort for evaluating the risk of in-hospital mortality in older patients with CAP. CURB-65: confusion, urea, respiratory rate, blood pressure, and age ≥ 65 years; qSOFA, quick Sequential Organ Failure Assessment; PSI, Pneumonia Severity Index.

In the training cohort, the C-index of the NB-DAPA nomogram was 0.90 (95% CI, 0.86–0.93), indicating good discernment. The Hosmer-Lemeshow test (χ^2^ = 4.19, *p* = 0.84) further proved excellent agreement between the predicted possibility and the actual observation for this nomogram (Figure 4A). Meanwhile, DCA revealed that the predictive model had significant net benefits for most threshold probabilities, suggestive of the potential clinical benefit of this predictive model (Figure 4B). Additionally, to test the feasibility of the application of our model in clinic, we compared the ROC of NB-DAPA nomogram model with PSI, CURB-65 and qSOFA, which have been commonly used in clinic and shown great predictive ability in CAP (18), to better explore the predictive function of mortality among elderly patients. The AUC values of NB-DAPA nomogram model, PSI, qSOFA and CURB-65 were 0.90 (95%CI: 0.86–0.93), 0.85 (95%CI: 0.81–0.87), 0.71 (95% CI: 0.66–0.76), 0.86 (95% CI: 0.83–0.90), respectively (Table 4). The analysis demonstrated that our model showed superiority when compared with PSI, CURB-65 or qSOFA, with NB-DAPA vs. PSI (*p* < 0.05, z = 6.79), vs. CURB-65 (*p* < 0.05, z = 2.08) and vs. qSOFA (*p* < 0.001, z = 6.55) (Table 5 and Figure 4C).

**TABLE 4:** The AUC of nomogram, PSI, CURB-65 and qSOFA for mortality in training cohort.

Variables	AUC	95% CI	*P*
Nomogram	0.90	0.86–0.93	<0.0001
qSOFA	0.71	0.66–0.76	<0.0001
PSI	0.85	0.81–0.87	<0.0001
CURB-65	0.86	0.83–0.90	<0.0001

AUC, area under the curve; CURB-65, confusion, urea, respiratory rate, blood pressure, and age ≥ 65 years; qSOFA, quick Sequential Organ Failure Assessment; PSI, Pneumonia Severity Index.

**TABLE 5:** Comparison of ROC for mortality in training cohort.

	Nomogram vs. qSOFA	Nomogram vs. CURB-65	Nomogram vs. PSI
Difference between areas	0.190	0.040	0.050
95% CI	0.131–0.243	0.002–0.069	0.138–0.249
z statistic	6.550	2.080	6.79
*P*-value	*p* < 0.0001	*p* < 0.05	*p* < 0.05

ROC, receiver operator characteristic curve; CURB-65, confusion, urea, respiratory rate, blood pressure, and age ≥ 65 years; qSOFA, quick Sequential Organ Failure Assessment; PSI, Pneumonia Severity Index.

**FIGURE 5:**
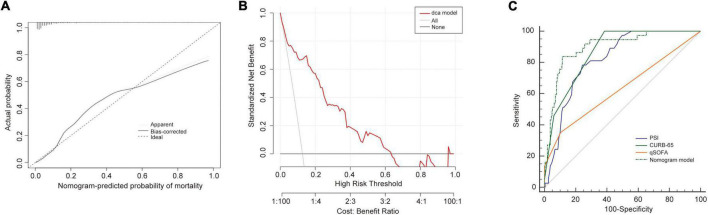
Validation of the nomogram in validation cohort set. **(A)** Calibration curves for predicting the mortality in the validation set. **(B)** Decision curve analysis for the nomogram model in the validation set. **(C)** The receiver operator characteristic curves (ROC) of the nomogram model, PSI, CURB-65 and qSOFA in validation cohort for predicting the risk of mortality in the elderly with CAP. CURB-65: confusion, urea, respiratory rate, blood pressure, and age ≥ 65 years; qSOFA, quick Sequential Organ Failure Assessment; PSI, Pneumonia Severity Index.

We then validated our model in the validation cohort. Accordingly, the C-index of the nomogram was 0.89 (95%CI: 0.84–0.95), supporting its good discriminative ability. Moreover, the calibration plots (χ^2^ = 12.69, *p* = 0.12) of the nomogram also revealed that the agreement between the predicted and observed disease severity was optimal in validation group (Figure 5A). Consistently, DCA plots conducted in validation sets also showed NB-DAPA nomogram model was associated with improved clinical net benefits with wide ranges of threshold probabilities (Figure 5B). Furthermore, we compared the ROC of nomogram model with PSI, CURB-65 or qSOFA in validation group. The AUC values of the nomogram model, PSI, qSOFA and CURB-65 were 0.89 (95%CI: 0.84–0.95), 0.82 (95%CI: 0.77–0.86), 0.62 (95% CI: 0.54–0.70), 0.84 (95% CI: 0.80–0.89), respectively (Table 6). Similarly, the results demonstrated NB-DAPA showed superiority to qSOFA (*p* < 0.001, z = 5.489) and PSI (*p* < 0.05, z = 1.96), even though no statistical significance was observed between the model and CURB-65 (*p* = 0.12, z = 1.54) (Table 7 and Figure 5C).

**TABLE 6:** The AUC of nomogram, PSI, CURB-65, and qSOFA for mortality in validation cohort.

Variables	AUC	95% CI	*P*
Nomogram	0.89	0.84–0.95	<0.0001
qSOFA	0.62	0.54–0.70	<0.0001
PSI	0.82	0.77–0.86	<0.0001
CURB-65	0.84	0.80–0.89	<0.0001

AUC, area under the curve; CURB-65, confusion, urea, respiratory rate, blood pressure, and age ≥ 65 years; qSOFA, quick Sequential Organ Failure Assessment; PSI, Pneumonia Severity Index.

**TABLE 7:** Comparison of ROC for mortality in validation cohort.

	Nomogram vs. qSOFA	Nomogram vs. CURB-65	Nomogram vs. PSI
Difference between areas	0.27	0.05	0.07
95% CI	0.175–0.370	0.013–0.112	0.001–0.157
z statistic	5.489	1.544	1.96
*P*-value	*p* < 0.0001	*p* = 0.12	*p* = 0.049

ROC, receiver operator characteristic curve; CURB-65, confusion, urea, respiratory rate, blood pressure, and age ≥ 65 years; qSOFA, quick Sequential Organ Failure Assessment; PSI, Pneumonia Severity Index.

### External validation

**FIGURE 6:**
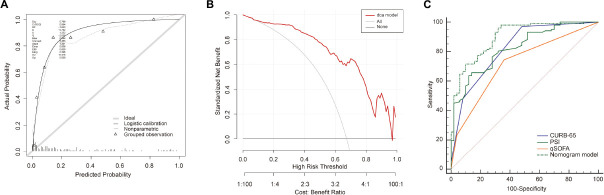
Validation of the nomogram in external validation cohort. **(A)** Calibration curves for predicting the mortality in the external validation set. **(B)** Decision curve analysis for the nomogram model in the external validation set. **(C)** The receiver operator characteristic curves (ROC) of the nomogram model, PSI, CURB-65 and qSOFA in external validation cohort for predicting the risk of mortality in the elderly with CAP. CURB-65: confusion, urea, respiratory rate, blood pressure, and age ≥ 65 years; qSOFA, quick Sequential Organ Failure Assessment; PSI, Pneumonia Severity Index.

We further recruited 153 elderly patients with CAP as an external validation cohort, and validated our model in this cohort. Consistently, the analysis in the external cohort showed the C-index of the nomogram was 0.89 (95%CI: 0.84–0.94), confirming its good discriminative ability. Moreover, the calibration plots (χ^2^ = 8, *p* = 0.29) and DCA plots of the nomogram reconfirmed that NB-DAPA nomogram model was a good predictive model (Figures 6A,B). Furthermore, we compared the ROC value among nomogram model, PSI, CURB-65 or qSOFA in external validation group. The results showed The AUC values of the nomogram model, PSI, qSOFA and CURB-65 were 0.89 (95%CI: 0.84–0.94), 0.82 (95%CI: 0.75–0.88), 0.72 (95% CI: 0.64–0.79), 0.82 (95% CI: 0.75–0.88), respectively (Table 8 and Figure 6C). Collectively, the ACU of nomogram showed the highest performance in the external validation group, which was in concert with the results from internal training and test cohorts.

**TABLE 8:** The AUC of nomogram, PSI, CURB-65, and qSOFA for mortality in external validation cohort.

Variables	AUC	95% CI	*P*
Nomogram	0.89	0.84–0.94	<0.0001
qSOFA	0.72	0.64–0.79	<0.0001
PSI	0.82	0.75–0.88	<0.0001
CURB-65	0.82	0.75–0.88	<0.0001

AUC, area under the curve; CURB-65, confusion, urea, respiratory rate, blood pressure, and age ≥ 65 years; qSOFA, quick Sequential Organ Failure Assessment; PSI, Pneumonia Severity Index.

## Discussion

This study enrolled 1,025 elderly patients with CAP from Minhang Hospital, Fudan University in Shanghai. We established and validated a nomogram for predicting the risk for mortality of CAP in elderly through LASSO and logistic regression analyses. Six variables have been identified to be independently statistically significant in the prediction model, including Age, Pulse, NLR, Serum Albumin, BUN, and D-dimer. Several statistical methods have also been used for the assessment of the model, suggestive of its optimal performance.

In this study, we showed several advantages. First, the population of enrolled patients is large and the outcomes in the training cohort is consistent with validation cohort. Second, our model is relatively simpler to use and consists of only six risk factors. Moreover, those variables can be obtained rapidly after admission in both primary and tertiary hospitals. Hence, our model is a fast assessment tool that can be used to prognosticate the severity of CAP in a variety of medical institutions. Third, the nomogram model is suitable for elderly patients with CAP.

In this population, our analysis demonstrated that NB-DAPA model was superior to PSI, CURB-65 and qSOFA. In details, the AUC of the nomogram model was about 0.9 for predicting in-hospital mortality of CAP and observed to be higher than PSI, CURB-65 and qSOFA values (0.85, 0.86, and 0.71, respectively). Among them, the evidence for the application of 4 selected variables (Age, Pulse, NLR, and BUN) in our model has been supported by various studies (19–23). Here, we also reported that the addition of serum albumin (ALB) and D-dimer can improve the predictive ability for mortality of CAP in patients aged ≥ 65 years. Moreover, common scoring systems of CAP severity such as CURB-65, qSOFA and PSI do not contain the two indicators and this may change the CAP severity scoring scales (24).

Our data showed the serum albumin is the highest scoring scale in nomogram model except for the BUN. We observed that the lower the concentration of serum albumin, the higher is mortality. And it decreased with aging for several potential reasons such as hepatic or renal disease and dysphagia, supported by some studies estimating albumin level reduction by 20% in patients over 70 years old (25). This observation is also supported by the evidence that hypoalbuminemia is associated with acquisition and severity of CAP (26, 27). In One study, Miyazaki et al. also showed that serum albumin is more important than CURB-65 or PSI class for predicting 30-day mortality in CAP patients (28). Recently, another study about predicting in-hospital mortality of CAP revealed that compared to patients with albumin ≥ 35 g/L, patients with albumin 25–34.9 g/L [OR = 4.929] and albumin < 25 g/L [OR = 12.463] were more likely to die at the time of hospitalization, but the predictive accuracy of albumin alone was similar to CURB-65 or PSI class (29). We speculate that this discrepancy could be explained as follows: First, low serum albumin levels are generally associated with severe inflammation and, most importantly, worse recovery (30). Second, the albumin molecule is central to its immunomodulatory effects, and maintains immune responses to invasive pathogens (31). Third, albumin oxidation and breakdown balance interactions with bio-active lipid mediators that play important roles in antimicrobial. Thus, hypoalbuminemia can result in a poor antimicrobial defense (32). Therefore, whether therapeutic strategy to increase the level of albumin (such as Human Albumin Infusion) could improve the prognosis of elderly patients with CAP requires to be further studied (32).

Another indicator of interests in our model is D-dimer. Here, higher elevated D-dimer level is associated with higher risk of mortality of patients with CAP. D-Dimer is a fibrin degradation product resulting from the sequential cleavage of the fibrinogen formed in the coagulation system by the fibrinolytic system (33). The procoagulant responses of the patient are closely related to inflammatory reaction to infection (34). During a systemic infection, sepsis-associated coagulopathy is characterized by concomitant activation of both extrinsic and contact coagulation, down-regulation of anticoagulants, and inhibition of fibrinolysis, finally leading to the generation of a variable amount of fibrin-related markers, including the D-dimer (35). Previously, some studies showed that the levels of D-dimer could be used to predict mortality in patients with CAP (36, 37). Further, D-dimer level < 500 ng/ml on admission has been associated with a lower risk of death and morbidity in CAP (38). Therefore, D-dimer seems to be an excellent biomarker in predicting prognosis of CAP. Additionally, D-dimer levels can be as a biomarker in patients with COVID-19 (39). Of note, although high D-dimer levels could indicate hypercoagulable state, it is not reasonable to judge whether anticoagulation is needed only according to D-dimer levels in CAP or COVID-19 (40). To the best of our knowledge, this is the first study to report that D-dimer levels could predict the mortality of elderly patients with CAP. More studies need to be performed to validate the relationship between CAP and D-dimer.

There are some limitations. First, this study is a single-center retrospective study based on a limited number of samples and the results of this study should be verified in multi-center, large-sample studies in the future. Second, in this study other clinical factors are not taken into consideration, such as antibiotic therapy and pathogen infection, which may also affect the prognosis of elderly patients with CAP (11). The last but not the least, published research showed that D-dimer levels increased with age (41, 42). So, in elderly patients, the D-dimer level should be adjusted by age. Currently, many studies have already reported the age-adjusted D-dimer cutoff values in elderly patients (43–45). However, there has been no such a concrete and unified calculation formula about the age-adjusted D-dimer.

## Conclusion

We established an early prediction model incorporating clinical characteristics that could be quickly obtained on hospital admission. This model can be conveniently used to predict the mortality of elderly patients with CAP. Moreover, the predictive performance of the NB-DAPA model was better than PSI, CURB-65 and qSOFA.

## Data availability statement

The original contributions presented in this study are included in the article/supplementary material, further inquiries can be directed to the corresponding author/s.

## Ethics statement

This study was approved by the Ethics Committee of the Minhang Hospital, Fudan University in Shanghai, and the Lot No: Medical Ethics Committee (2019) No. 50. The patients/participants provided their written informed consent to participate in this study.

## Author contributions

CL and JD: conceptualization. CL and ML: methodology and formal analysis. ML: software. ML, WS, and TP: validation. CL, ML, and JD: investigation. CL and JX: resources and data curation. CL, AM, and WP: writing—original draft preparation. JD and JX: writing—review and editing and supervision. CL, WS, and TP: visualization. CL: project administration. All authors have read and agreed to the published version of the manuscript, contributed to the article, and approved the submitted version.
